# MTBP inhibits the Erk1/2-Elk-1 signaling in hepatocellular carcinoma

**DOI:** 10.18632/oncotarget.25117

**Published:** 2018-04-20

**Authors:** Atul Ranjan, Swathi V. Iyer, Christopher Ward, Tim Link, Francisco J. Diaz, Animesh Dhar, Ossama W. Tawfik, Steven A. Weinman, Yoshiaki Azuma, Tadahide Izumi, Tomoo Iwakuma

**Affiliations:** ^1^ Department of Cancer Biology, University of Kansas Medical Center, Kansas City, KS, USA; ^2^ Department of Biostatistics, University of Kansas Medical Center, Kansas City, KS, USA; ^3^ Department of Pathology, University of Kansas Medical Center, Kansas City, KS, USA; ^4^ Department of Internal Medicine, University of Kansas Medical Center, Kansas City, KS, USA; ^5^ Department of Molecular Bioscience, University of Kansas, Lawrence, KS, USA; ^6^ Department of Toxicology and Cancer Biology, University of Kentucky College of Medicine, Lexington, KY, USA; ^7^ Children's Research Institute, Children's Mercy Hospital and Clinics, Kansas City, MO, USA

**Keywords:** MDM2, MTBP, Erk1/2, Elk-1, metastasis

## Abstract

Hepatocellular carcinoma (HCC) is one of the most common cancers worldwide, and the prognosis of HCC patients, especially those with metastasis, remains extremely poor. This is partly due to unclear molecular mechanisms underlying HCC metastasis. Our previous study indicates that MDM2 Binding Protein (MTBP) suppresses migration and metastasis of HCC cells. However, signaling pathways regulated by MTBP remain unknown. To identify metastasis-associated signaling pathways governed by MTBP, we have performed unbiased luciferase reporter-based signal array analyses and found that MTBP suppresses the activity of the ETS-domain transcription factor Elk-1, a downstream target of Erk1/2 MAP kinases. MTBP also inhibits phosphorylation of Elk-1 and decreases mRNA expression of Elk-1 target genes. Reduced Elk-1 activity is caused by inhibited nuclear translocation of phosphorylated Erk1/2 (p-Erk) by MTBP and subsequent inhibition of Elk-1 phosphorylation. We also reveal that MTBP inhibits the interaction of p-Erk with importin-7/RanBP7 (IPO7), an importin family member which shuttles p-Erk into the nucleus, by binding to IPO7. Moreover, high levels of MTBP in human HCC tissues are correlated with cytoplasmic localization of p-Erk1/2. Our study suggests that MTBP suppresses metastasis, at least partially, by down-modulating the Erk1/2-Elk-1 signaling pathway, thus identifying a novel regulatory mechanism of HCC metastasis by regulating the subcellular localization of p-Erk.

## INTRODUCTION

Metastasis is the primary cause of the poor prognosis in cancer patients, being responsible for about 90% of deaths of patient with solid tumors [1]. Migration of cancer cells to distant sites in the body is one of the critical steps for metastasis [2]. Identifying the molecular mechanisms of cancer cell migration and metastasis will significantly help in improvement of prognosis of cancer patients.

Hepatocellular carcinoma (HCC) is the third most common cause of cancer-related death and stands fifth in ranking of most common cancer worldwide. The 5-year survival rate is extremely poor despite surgical resection. This is mainly due to microvascular invasion and extrahepatic metastasis to lymph nodes, lungs, and bones [3, 4]. Hence, identifying and characterizing critical molecular mediators of metastasis suppression would lead to therapeutic intervention in HCC precision therapy.

MDM2 Binding Protein (MTBP) was initially identified as a protein that interacted with the oncoprotein Mouse Double Minute 2 homolog (MDM2) [5]. Increasing evidence suggests that MTBP inhibits cancer cell migration and metastasis independent of the MDM2-p53 pathway, since MTBP knockdown increases migration of cells lacking both p53 and MDM2 [6, 7]. In head and neck carcinoma, reduced MTBP expression in tumors is correlated with reduced patient survival [8]. We have previously reported that MTBP expression is reduced in HCC tissues while overexpression of MTBP inhibits cancer cell migration in osteosarcoma and HCC in a p53-independent manner in culture and in mouse models [7, 9]. On the other hand, there are a few reports suggesting cancer-promoting roles of MTBP in leukemia and breast cancer by cooperating with Myc [10–12]. Thus, the role of MTBP in cancer progression appears to be dependent on cellular context [6]. Additionally, other functions of MTBP have been reported; these include its roles in chromosome segregation [13] and DNA replication origin firing [14].

Our recent study suggests that MTBP suppresses migration of HCC cells, at least partially, by inhibiting the actin-crosslinking function of α-actinin-4 (ACTN4) [9]. However, the mechanism behind MTBP-mediated suppression of HCC migration other than ACTN4 inhibition remains unknown. Toward this goal and to understand signaling pathways involved in MTBP-mediated migration suppression in HCC, we performed an unbiased screening using a luciferase-based signal array with two HCC cell lines, leading to identification of MTBP as a negative regulator of the Erk1/2-Elk-1 signaling pathway. This effect of MTBP was indirectly caused by its inhibitory interaction with importin-7/RanBP7 (IPO7), a key protein that imports phosphorylated Erk1/2 (p-Erk) from the cytoplasm to the nucleus.

## RESULTS

### MTBP inhibits Elk-1 activity

**Figure 1:**
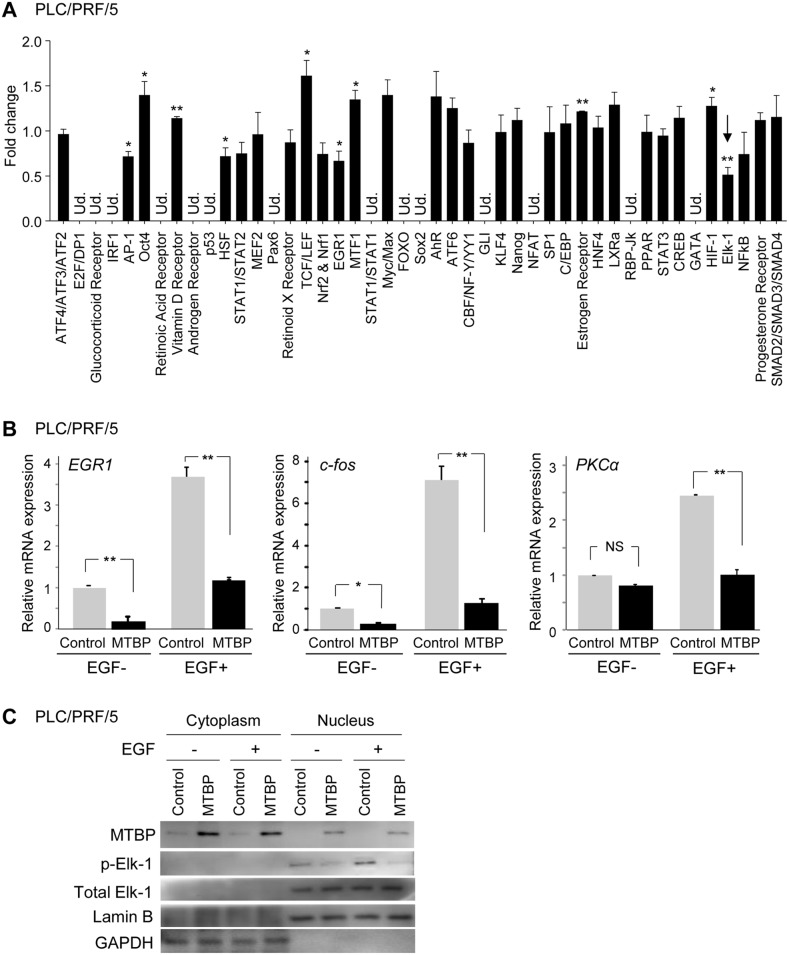
MTBP inhibits Elk-1 activity **(A)** Luciferase-based signal array analysis using PLC/PRF/5 cells infected with lentiviral vectors encoding empty (control) or MTBP cDNA. Results showing fold change of the luciferase activity altered by MTBP overexpression, compared to the control lentiviral vector (set as 1) of 45 genes related with different signaling pathways. An arrow indicates that Elk-1 activity was significantly suppressed by MTBP overexpression. Ud., undetectable. Error bars: mean ± S.D. from three independent experiments. Student's *t* test: ^*^, P < 0.05 and ^**^, P < 0.01. Other pathways had no statistical significance. **(B)** Results of qRT-PCR to measure mRNA expression of *EGR1*, *c-fos*, and *PKCα* using PLC/PRF/5 cells infected with lentiviral vectors encoding empty (control, grey) or MTBP cDNA (black), with (+) or without (−) 50 ng/ml of EGF stimulation for 15 min. Data are normalized by values of *GAPDH* mRNA. Error bars: means ± S.D. from three independent experiments. Student's *t* test: ^*^, P < 0.05 and ^**^, P < 0.01. NS, not significant at a 0.05 significance level. **(C)** Western blotting for MTBP, phosphorylated Elk-1 at serine 383 (p-Elk-1), total Elk-1, Lamin B, and GAPDH using cytoplasmic and nuclear protein extracts from PLC/PRF/5 cells treated with solvent (−) or 50 ng/ml of EGF (+) for 30 min.

To understand the molecular basis of the biological function of MTBP and determine signaling pathways regulated by MTBP, we performed unbiased signal array analyses using the luciferase reporter-based Cignal Finder Reporter Arrays (SA Biosciences). This assay allows measuring activities of 45 transcription factors associated with specific signaling pathways. Since MTBP's effects on cancer cell migration is independent of the MDM2-p53 pathway [6, 7], we performed luciferase assays using mutant p53-carrying HCC cells lines, PLC/PRF/5 (p53^R249S^) and Huh7 (p53^Y220C^), and the results were compared between control cells and those overexpressing MTBP. In both cell lines tested, the activity of an ETS-domain transcription factor Elk-1 was consistently suppressed by MTBP overexpression up to ~45% when compared to controls (Figure 1A and Supplementary Figure 1A). AP-1 and EGR1 transcriptional activities were also suppressed by MTBP to a lesser extent. Since both transcription factors are downstream targets of Elk-1, this could be due to reduced Elk-1 activity [15, 16]. In contrast with previous reports using lymphoma or MCF7 cells [10, 17], activity of c-Myc was not significantly altered by MTBP in PLC/PRF/5 and Huh7 cells (Figure 1A and Supplementary Figure 1A). This divergence may be due to the differences in tissue types or cellular contexts.

Elk-1 is phosphorylated and activated as a transcription factor following treatment with several growth factors, such as EGF [18]. To validate inhibition of Elk-1 activity by MTBP, we performed quantitative RT-PCR (qRT-PCR) to measure mRNA expression of three Elk-1 target genes, *EGR-1, c-fos,* and *PKCα,* using PLC/PRF/5 and Huh7 cell lines with or without overexpression of MTBP, in the absence or presence of EGF stimulation [18]. In agreement with signal array analyses, MTBP overexpression significantly suppressed mRNA induction of all the tested Elk-1 target genes by EGF (Figure 1B and Supplementary Figure 1B).

To further confirm that MTBP inhibited activation of Elk-1, phosphorylation status of Elk-1 was examined using cytoplasmic and nuclear protein extracts. EGF treatment increased phosphorylation of Elk-1 in the nucleus which was significantly inhibited by MTBP overexpression in both PLC/PRF/5 and Huh7 cells (Figure 1C and Supplementary Figure 1C). These results suggest that MTBP suppresses Elk-1 transcriptional activity through inhibition of Elk-1 phosphorylation.

### MTBP inhibits nuclear translocation of phosphorylated Erk1/2

**Figure 2:**
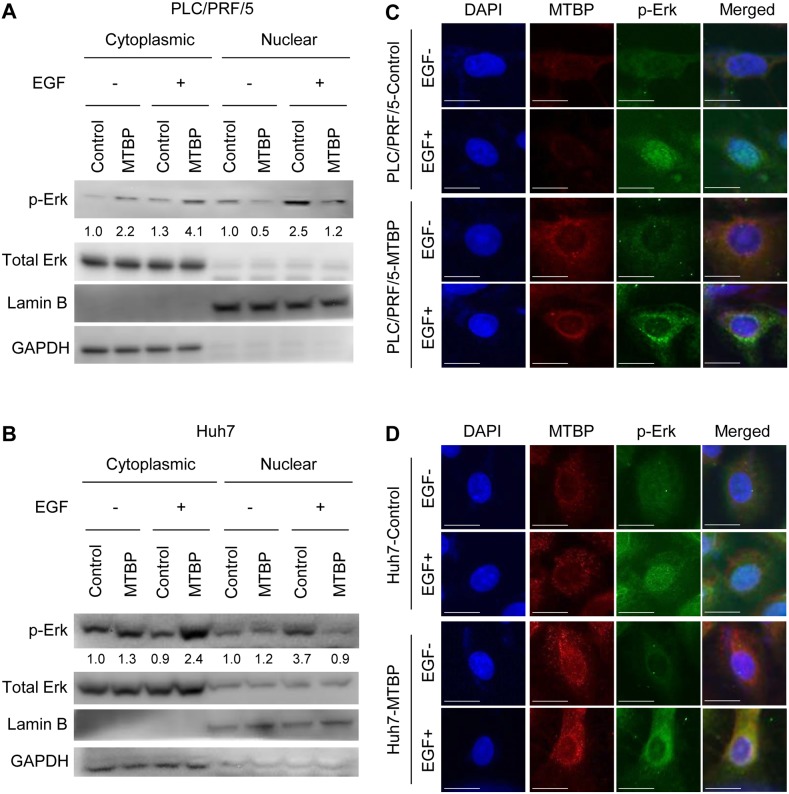
MTBP inhibits nuclear translocation of phosphorylated Erk1/2 **(A and B)** Western blotting for Erk1/2 and phosphorylated Erk1/2 at Thr202/Tyr204 (p-Erk) using cytoplasmic and nuclear protein extracts from PLC/PRF/5 (A) and Huh7 (B) cells treated with solvent (−) or 50 ng/ml of EGF (+) for 30 min. By setting the control cytoplasmic fraction and the control nuclear fraction as 1, the relative ratios for p-Erk/total Erk to the control fractions are shown below p-Erk blots. **(C and D)** Immunofluorescence studies for DAPI (blue), MTBP (red), and p-Erk (green) following treatment of control or MTBP-overexpressing cells with (+) or without (−) 50 ng/ml of EGF treatment for 30 min in PLC/PRF/5 (C) and Huh7 (D) cells. Merged images for DAPI, MTBP, and p-Erk are also shown on the right. Scale bar, 25 μm.

Phosphorylation of Elk-1 in the nucleus is mediated by phosphorylated Erk1/2 MAP kinase (p-Erk), leading to transactivation of downstream target genes involved in cell migration and metastasis [18–21]. Hence, we investigated whether MTBP could modulate the activity of Erk1/2 by examining phosphorylation status and subcellular localization of Erk1/2. Results from western blotting showed that MTBP overexpression caused increase (approximately 2- to 3-fold) in the cytoplasmic p-Erk with decrease (approximately 2- to 4-fold) in the nuclear p-Erk when compared to the controls, especially upon EGF treatment, suggesting inhibition of p-Erk nuclear import by MTBP (Figure 2A and 2B). Notably, overall cellular amount (nucleus plus cytoplasm) of p-Erk was similar between control and MTBP-overexpressing cells (Figure 2A and 2B). Immunofluorescence studies also revealed that EGF-induced nuclear import of p-Erk was inhibited in both PLC/PRF/5 and Huh7 cells overexpressing MTBP when compared with control cells (Figure 2C and 2D). These results suggest that MTBP inhibits Elk-1 activity mainly by preventing nuclear translocation of p-Erk, rather than altering phosphorylation status of Erk1/2.

MTBP is shown to alter cancer cell migration and metastasis regardless of p53 status. Since we used HCC cells with p53 mutations, we additionally examined whether MTBP altered nuclear translocation of p-Erk upon EGF stimulation in HepG2 cells expressing wild-type p53. As expected, MTBP overexpression still inhibited nuclear translocation of p-Erk in HepG2 cells (Supplementary Figure 2A and 2B). We furthermore assessed whether MTBP inhibited mRNA expression of Elk-1 target genes (*EGR-1, c-fos, PKCα*) in HepG2 cells. Consistently, MTBP overexpression suppressed EGF-mediated increase in mRNA expression of Elk-1 target genes, as well as their basal expression levels without EGF treatment (Supplementary Figure 2C). Together, these results strongly suggest that MTBP inhibits the Erk1/2-Elk-1 signaling regardless of the p53 status.

### MTBP inhibits HCC cell migration through suppressing the Erk1/2-Elk-1 axis

**Figure 3:**
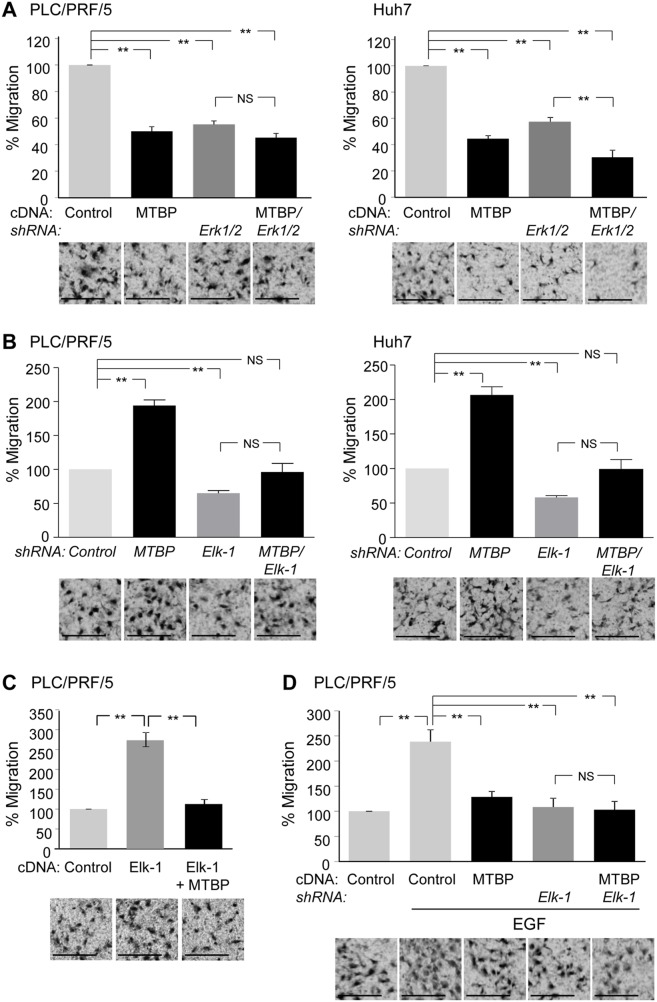
MTBP inhibits HCC cell migration through suppressing the Erk1/2-Elk-1 axis **(A)** Transwell migration assays using PLC/PRF/5 (left) and Huh7 (right) cells downregulated for Erk1/2 with or without overexpression of MTBP. Cells infected with lentiviral vectors encoding a non-target shRNA (control) or shRNAs for Erk1 and Erk2 (*Erk1/2*) were further infected with a lentiviral vector encoding MTBP cDNA (MTBP). Cells were plated on the upper chambers of the transwell, and migrating cells in the entire fields were counted 12 hours later. **(B)** Transwell migration assays using PLC/PRF/5 (left) and Huh7 (right) cells knockdown for Elk-1 (*Elk-1*) and/or MTBP (*MTBP*). **(C)** Transwell migration assays using PLC/PRF/5 cells infected with lentiviral vectors encoding empty (control) or Elk-1 cDNA (Elk-1) with or without concomitant overexpression of MTBP. **(D)** Transwell migration assays using PLC/PRF/5 cells infected with lentiviral vectors encoding non-target (control) or *Elk-1* shRNAs with or without concomitant overexpression of MTBP, in the presence of EGF (100 ng/ml). Graphs showing relative cell migration (%) compared to the number of migrating cells in control. Representative images below the graphs. Error bars: means ± S.D. from three independent experiments. Student's *t* test: ^**^, P < 0.01. NS, not significant. Scale bar, 25 μm.

Next, we wanted to examine the functional association between MTBP and the Erk1/2-Elk-1 axis on HCC cell migration using transwell migration assays. First, MTBP overexpression or knockdown of Erk1/2 significantly reduced migratory potential of both PLC/PRF/5 and Huh7 cells (Figure 3A and western blotting results in Supplementary Figure 3A). Concomitant overexpression of MTBP in Erk1/2-knockdown cells resulted in further reduction in migration of Huh7 cells, but there was no significant reduction in PLC/PRF/5 cells (Figure 3A and western blotting results in Supplementary Figure 3A). Since our previous study shows that migration of Huh7 cells is partially dependent on ACTN4, but migration of PLC/PRF/5 cells is ACTN4-independent [9], these data may suggest that migratory potential of PLC/PRF/5 is mainly dependent on the Erk1/2-Elk-1 axis.

We next examined functional association between MTBP and Elk-1. Increased migration by MTBP knockdown was attenuated by concomitant knockdown of Elk-1 in both PLC/PRF/5 and Huh7 cell lines (Figure 3B and western blotting results in Supplementary Figure 3B). Also, overexpression of Elk-1 in PLC/PRF/5 cells increased the migration, which was nullified by concomitant overexpression of MTBP (Figure 3C and western blotting results in Supplementary Figure 3C). Moreover, overexpression of MTBP in Elk-1-knockdown cells failed to further reduce migration of PLC/PRF/5 cells in the presence of EGF (Figure 3D and western blotting results in Supplementary Figure 3D). These results suggest that migration suppression by MTBP in PLC/PRF/5 cells is mainly dependent on the Erk1/2-Elk-1 axis. On the other hand, overexpression of MTBP in Elk-1-knockdown Huh7 cells still reduced cell migration (Supplementary Figure 3D and 3E). These results support the idea that MTBP inhibits migration of Huh7 cells in Erk1/2-Elk-1 axis–dependent and –independent manners.

We also examined whether MTBP could inhibit migration of wild-type p53-expressing HepG2 cells via Elk-1. As expected, MTBP knockdown increased migration of HepG2 cells, which was significantly attenuated by concomitant knockdown of Elk-1 (Supplementary Figure 4A and 4B). Additionally, we tested whether mutant p53 was involved in the MTBP-mediated inhibition of Elk-1 and HCC cell migration, using Huh7 cells down-modulated for mutant p53. Although knockdown of mutant p53 decreased migration of Huh7 cells, knockdown of MTBP in mutant p53-knockdown Huh7 cells still increased the migration, which was significantly attenuated by simultaneous knockdown of Elk-1 (Supplementary Figure 4C and 4D). Thus, MTBP inhibits HCC cell migration, at least partially, by inhibiting Elk-1 activity, regardless of the p53 status.

### MTBP inhibits interactions between p-Erk and IPO7 by binding to IPO7

**Figure 4:**
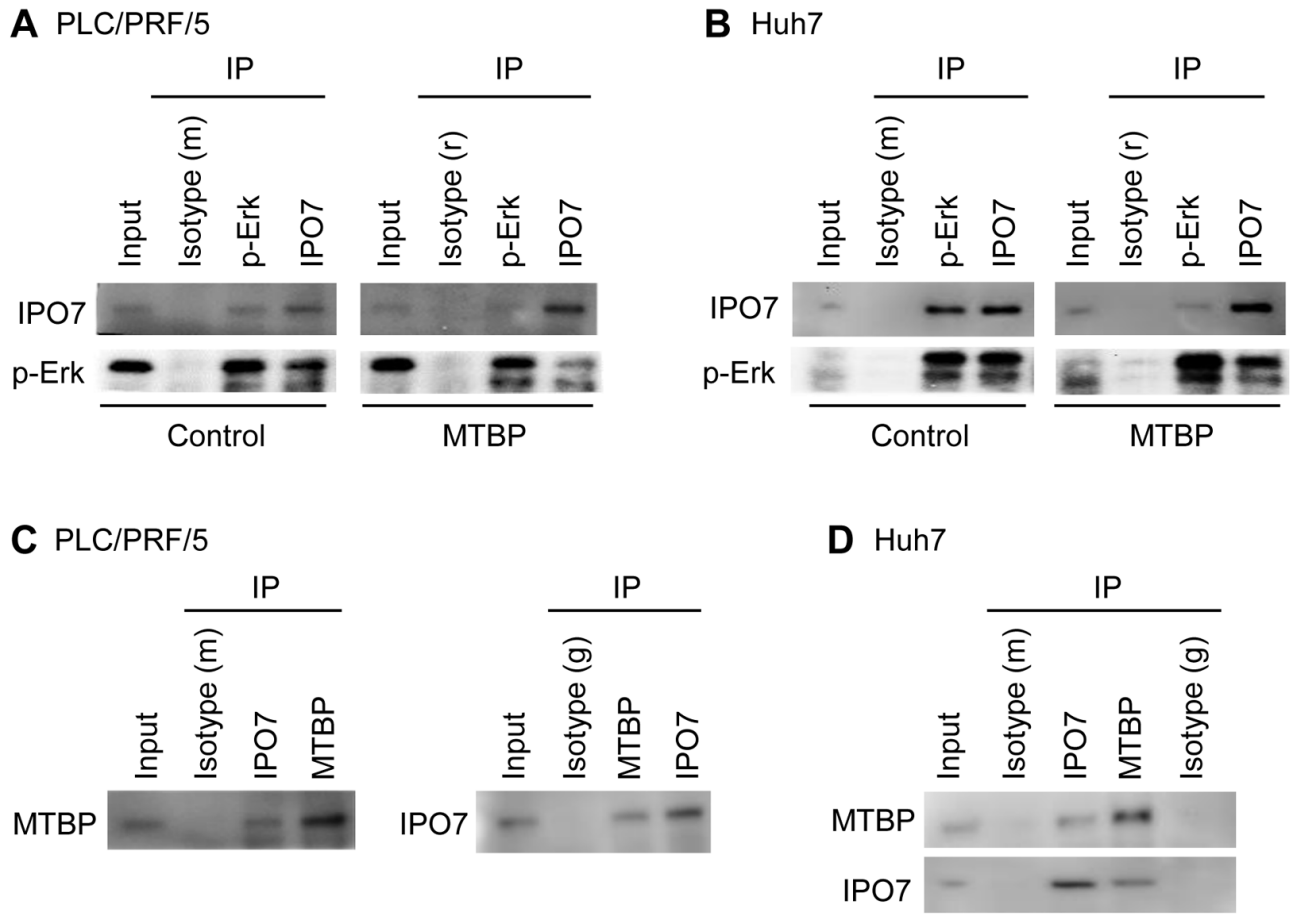
MTBP inhibits interactions between p-Erk and IPO7 by binding to IPO7 **(A and B)** Co-immunoprecipitation (IP) studies for endogenous p-Erk and IPO7 using protein extracts (~200 μg) from PLC/PRF/5 (A) and Huh7 (B) cells infected with lentiviral vectors encoding empty (control) or MTBP cDNA (MTBP). Isotypes were used as negative controls (m: mouse, r: rabbit). **(C and D)** Co-immunoprecipitation studies for endogenous MTBP and IPO7 using PLC/PRF/5 (C) and Huh7 (D) cells. Isotypes were used as negative controls (m: mouse, g: goat). 10% of the total amount of protein lysate (~20 μg) was used for input.

Neither Erk1 nor Erk2 has a nuclear localization signal (NLS) [22, 23], while importin 7 (IPO7) is the major player that shuttles p-Erk into the nucleus [24, 25]. We therefore examined the effects of MTBP overexpression on the interactions between p-Erk and IPO7 by co-immunoprecipitation studies using both PLC/PRF/5 and Huh7 cells. In both cell lines, overexpression of MTBP inhibited the endogenous interaction between p-Erk and IPO7 (Figure 4A and 4B; lane 3 vs lane 7 for IPO7 blots, lane 4 and lane 8 for p-Erk blots).

To determine whether MTBP could endogenously interact with p-Erk and/or IPO7, co-immunoprecipitation studies were performed using protein extracts from PLC/PRF/5 and Huh7 cells. Endogenous interactions between MTBP and IPO7 were detected in both cell lines (Figure 4C and 4D). However, we failed to observe the MTBP-p-Erk interaction in both cell lines (Supplementary Figure 5). These results propose that MTBP competitively binds with IPO7 to inhibit the p-Erk-IPO7 interaction, leading to cytoplasmic retention of p-Erk and hence attenuated Elk-1 phosphorylation and activity.

### C-terminal region of MTBP is required for binding to IPO7 and inhibiting p-Erk nuclear translocation

**Figure 5:**
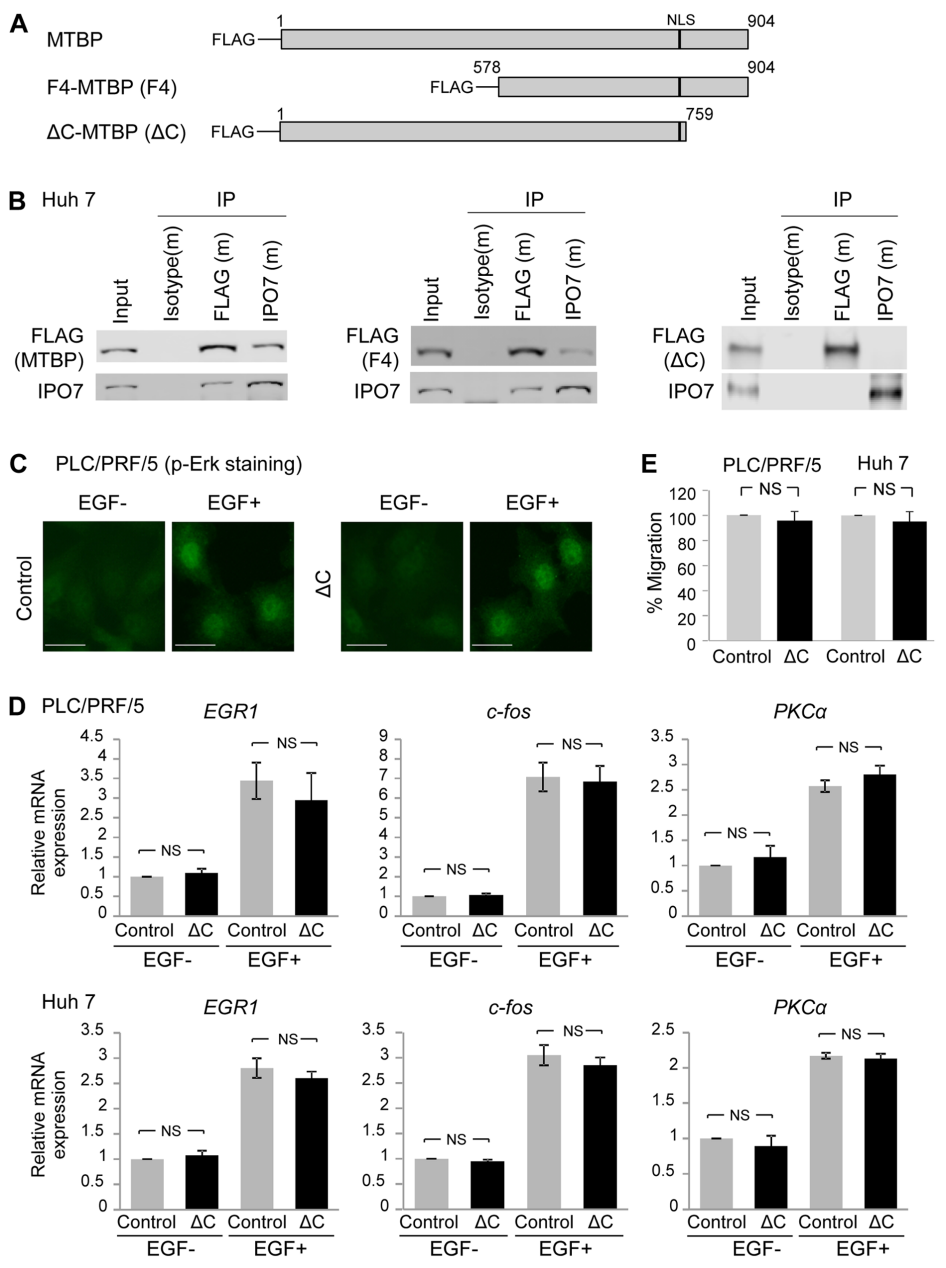
C-terminal region of MTBP is required for binding to IPO7 and inhibiting p-Erk nuclear translocation **(A)** Deletion constructs of MTBP tagged with the FLAG peptide at the N-terminus. MTBP: full-length MTBP; F4-MTBP (F4): N-terminal region deleted MTBP; ΔC-MTBP (ΔC): C-terminal region deleted MTBP. Numbers indicate amino acid of MTBP. Black bars indicate a nuclear localization signal (NLS) spanning between codons 730 and 753. **(B)** Co-immunoprecipitation (IP) studies between MTBP (FLAG tag) and IPO7 using protein extracts (~200 μg) from Huh7 cells expressing full-length MTBP (MTBP), F4-MTBP (F4), and ΔC-MTBP (ΔC). Isotypes were used as negative controls (m: mouse). 10% of the total amount of protein lysate (~20 μg) was used for input. **(C)** Immunofluorescence studies for p-Erk following treatment with solvent (EGF-) or 50 ng/ml of EGF for 30 min using PLC/PRF/5 cells with (ΔC) or without (control) overexpression of ΔC-MTBP. Scale bar, 50 μm. **(D)** Results of qRT-PCR to measure mRNA expression of *EGR1*, *c-fos*, and *PKCα* using PLC/PRF/5 (top) and Huh7 (bottom) cells infected with lentiviral vectors encoding empty (control, grey) or ΔC-MTBP cDNA (ΔC, black), with (+) or without (−) 50 ng/ml of EGF stimulation for 15 min. Data are normalized by values of *GAPDH* mRNA. Error bars: means ± S.D. from three independent experiments. Student's *t* test. NS, not significant. **(E)** Transwell migration assays for 12 hours using PLC/PRF/5 (left) and Huh7 (right) cells infected with lentiviral vectors encoding empty (control) and ΔC-MTBP cDNA (ΔC). Error bars: means ± S.D. from three independent experiments. Student's *t* test. NS, not significant.

Next, we attempted to identify a region required for the MTBP-IPO7 interaction. We previously generated a series of N-terminal FLAG-tagged deletion/mutation constructs for MTBP, including full-length MTBP (MTBP), N-terminal region deleted mutant (F4-MTBP), and C-terminal region deleted mutant (ΔC-MTBP, Figure 5A) [7]. We overexpressed these FLAG-tagged MTBP in Huh7 cells and performed co-immunoprecipitation studies with FLAG- and IPO7-specific antibodies. We found that full-length and F4-MTBP interacted with endogenous IPO7, but ΔC-MTBP failed to bind with IPO7 (Figure 5B). Thus, the C-terminal region spanning from amino acids 760 to 904 of MTBP was required for the MTBP-IPO7 interaction (Figure 5B).

We also examined whether the ΔC-MTBP retained the ability to inhibit the Erk1/2-Elk-1 signaling. As expected, overexpression of ΔC-MTBP which could not bind to IPO7 failed to inhibit nuclear translocation of p-Erk, following EGF stimulation in PLC/PRF/5 cells (Figure 5C). Also, qRT-PCR assays to measure mRNA expression of the Elk-1 target genes, *EGR-1, c-fos*, and *PKCα* revealed that ΔC-MTBP lost the ability to suppress mRNA expression of Elk-1 downstream targets, unlike the case of full-length MTBP (Figure 5D, compare the result with Figure 1A) in both PLC/PRF/5 and Huh7 cells. Moreover, unlike full-length MTBP, overexpression of ΔC-MTBP failed to reduce the migration of both PLC/PRF/5 and Huh7 cells (Figure 5E). These results suggest that the C-terminal region of MTBP is required for binding to IPO7, inhibiting the Erk1/2-Elk-1 signaling, and suppressing HCC migration.

### Cytoplasmic MTBP plays a role in inhibiting the Erk1/2-Elk-1 signaling

**Figure 6:**
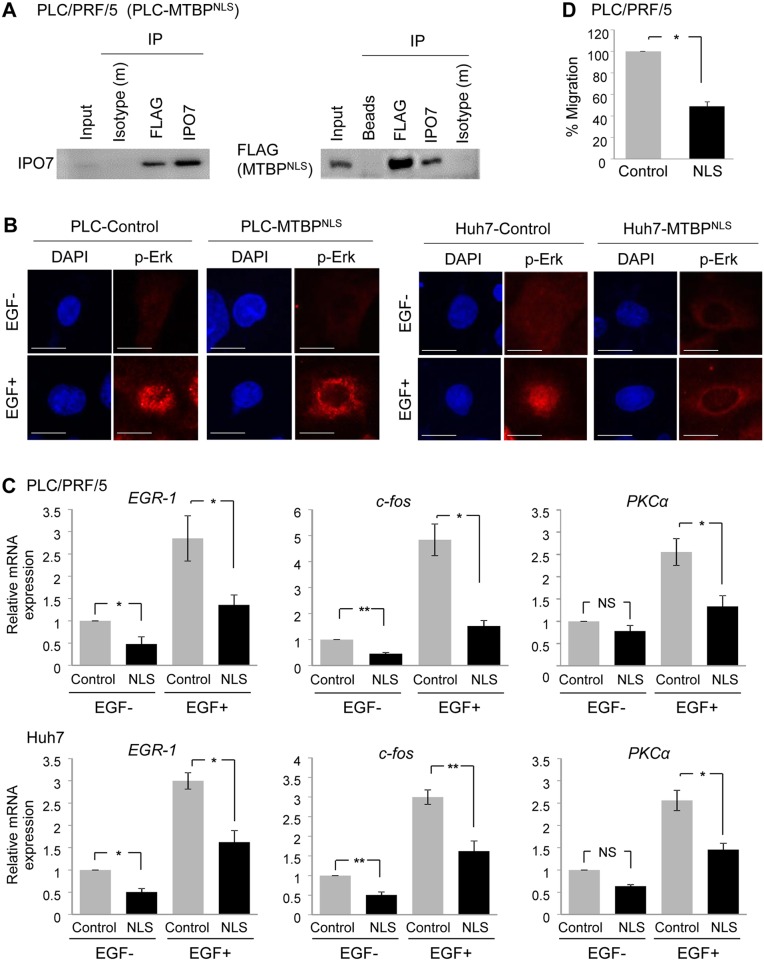
Cytoplasmic MTBP plays a role in inhibiting the Erk1/2-Elk-1 signaling **(A)** Co-immunoprecipitation studies between a NLS mutant MTBP (MTBP^NLS^) and IPO7 using protein lysates (~200 μg) from PLC/PRF/5 cells expressing FLAG-tagged MTBP^NLS^. MTBP^NLS^ was precipitated using anti-FLAG M2 affinity gel. Isotypes and protein A/G agarose beads were used as negative controls. 10% of the total amount of protein lysate (~20 μg) was used for input. **(B)** Immunofluorescence studies for p-Erk following treatment with solvent (EGF-) or 50 ng/ml of EGF for 30 min using PLC/PRF/5 (left) and Huh7 (right) cells with (MTBP^NLS^) or without (control) overexpression of MTBP^NLS^. Scale bar, 25 μm. **(C)** Results of qRT-PCR to measure *EGR1*, *c-fos*, and *PKCα* mRNA expression using PLC/PRF/5 (top) and Huh7 (bottom) cells infected with lentiviral vectors encoding empty (control, grey) or MTBP^NLS^ cDNA (NLS, black), with (+) or without (−) 50 ng/ml of EGF stimulation for 15 min. Data are normalized by values of *GAPDH* mRNA. Error bars: means ± S.D. from three independent experiments. ^*^, P < 0.05 and ^**^, P < 0.01; Student's *t* test. NS, not significant. **(D)** Transwell migration assays for 12 hours using PLC/PRF/5 cells infected with lentiviral vectors encoding empty (control) or FLAG-tagged MTBP^NLS^. Graphs showing relative cell migration (%) compared to the number of migrating cells in control. Error bars: means ± S.D. from three independent experiments. ^*^, P < 0.05; Student's *t* test.

MTBP is present in both the cytoplasm and nucleus [7]. We therefore wanted to examine whether the cytoplasmic portion of MTBP played a role in inhibiting IPO7's ability to import p-Erk to the nucleus. MTBP possesses a NLS which is required for MTBP protein to be imported into the nucleus (Figure 5A). First, we confirmed that a mutant MTBP with disruption of the NLS (MTBP^NLS^) [7] retained the ability to bind with IPO7 in PLC/PRF/5 and Huh7 cells by co-immunoprecipitation studies for FLAG-tagged MTBP^NLS^ and IPO7 (Figure 6A and Supplementary Figure 6A). Next, we performed immunofluorescence studies for p-Erk using both PLC/PRF/5 and Huh7 cells with or without overexpression of FLAG-tagged MTBP^NLS^ and in the absence or presence of EGF stimulation. In both cell lines, MTBP^NLS^ still inhibited the nuclear translocation of p-Erk following EGF stimulation (Figure 6B). Also, we examined whether MTBP^NLS^ retained the ability to suppress Elk-1 activity by measuring transcription of Elk-1 target genes in both PLC/PRF/5 and Huh7 cells (Figure 6C). As expected, MTBP^NLS^ inhibited mRNA expression of Elk-1 downstream targets with or without EGF stimulation, as in the case of full-length MTBP (Figure 6C, compare the result with Figure 1B). Moreover, overexpression of MTBP^NLS^ reduced the migration of PLC/PRF/5 and Huh7 cells (Figure 6D and Supplementary Figure 6B). These results suggest that the cytoplasmic portion of MTBP plays a crucial role in inhibiting the Erk1/2-Elk-1 signaling and migration.

### High MTBP levels are correlated with cytoplasmic p-Erk in human HCC tissues

**Figure 7:**
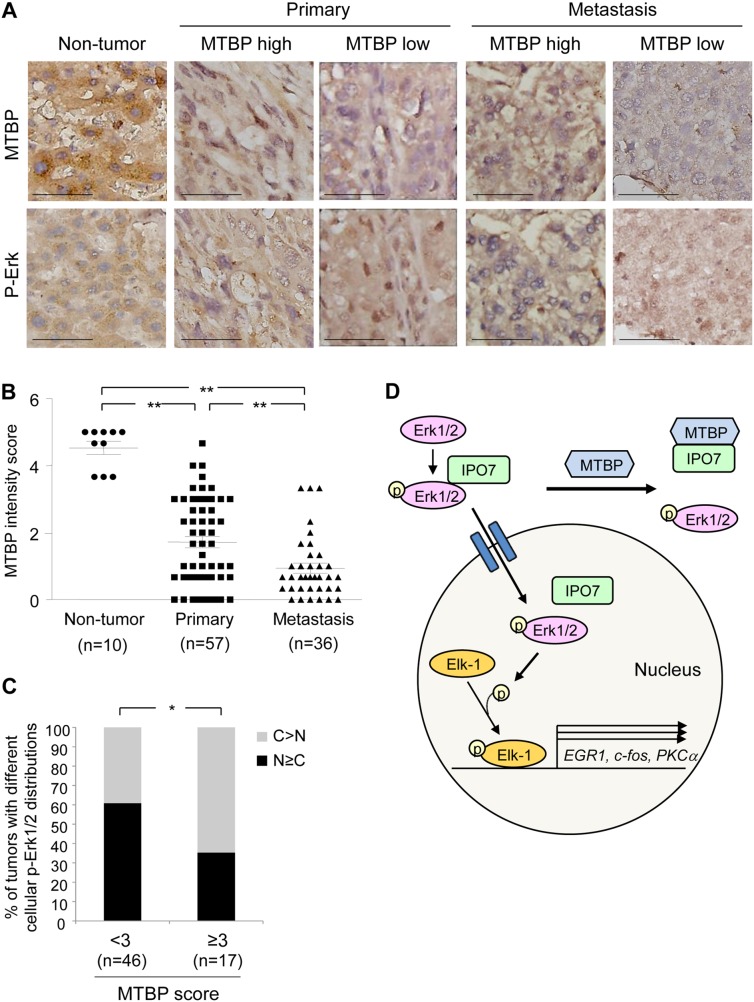
High MTBP levels are correlated with cytoplasmic p-Erk in human HCC tissues **(A)** Representative images of IHC for MTBP and p-Erk using human non-tumor liver tissues (n=10), primary HCC (n= 57), and metastatic HCC (n= 36) tissues. MTBP high: score≥3, MTBP low: score<3. Scale bar, 25 μm. **(B)** Summary of IHC for MTBP. Graph showing MTBP expression (immunoreactive) scores in non-tumor liver, primary HCC, and metastatic HCC tissues. Mann-Whitney test: ^**^, P < 0.01. **(C)** Summary of association between MTBP levels and subcellular localization of p-Erk in human HCC tissues (combining primary and metastatic tissues). Graph showing % of tumors with different cellular distributions of p-Erk by expression scores of MTBP (<3 vs ≥3). Exact logistic regression controlling for tumor location: ^*^, P < 0.05. **(D)** Proposed model for MTBP-mediated inhibition of the Erk1/2-Elk-1 signaling pathway. MTBP inhibits binding of p-Erk with IPO7, which reduces nuclear import of p-Erk, leading to decrease in Elk-1 phosphorylation and Elk-1 downstream target gene expression (*EGR1*, *c-fos*, *PKCα*).

Our tissue culture experiments indicate that MTBP negatively regulates nuclear import of p-Erk. To investigate correlation between MTBP levels and p-Erk subcellular localization in human HCC tissues, we performed immunohistochemistry (IHC) for MTBP and p-Erk using paraffin embedded non-tumor liver and HCC tissues, comprising 10 non-tumor liver, 57 primary HCC, and 36 metastatic HCC tissues. MTBP was diffusively located to both the cytoplasm and the nucleus in non-tumor liver and HCC tissues, while the staining patterns of p-Erk were diverse where some were localized to the nucleus and others were mainly in the cytoplasm (Figure 7A). Consistent with our previous report [9], overall MTBP levels were significantly lower in both primary and metastatic HCC tumors when compared with non-tumor liver tissues, although there were some HCC tissues showing moderately high levels of MTBP (Figure 7B). Interestingly, MTBP levels in HCC at metastatic sites were significantly lower than those at primary sites (Figure 7B).

To test the hypothesis that high levels of MTBP are associated with increased cytoplasmic p-Erk in HCC tissues, we scored subcellular localization of p-Erk as cytoplasm (C)>nucleus (N) vs C≤N, along with MTBP expression levels which were categorized as low (<3) vs high (≥3) scores, using both primary and metastatic HCC tissues. Interestingly, there was a significant association between high expression of MTBP and cytoplasmic localization of p-Erk after controlling for tumor location (Figure 7A, 7C, and Supplementary Table 1; p-value from exact logistic regression=0.034). We could not detect significant association of levels of MTBP or p-Erk with tumor size or pathological grade of primary HCC (Supplementary Table 2). These results support our tissue culture data showing that MTBP inhibits nuclear import of p-Erk1/2.

Sorafenib is a multikinase inhibitor that is shown to improve survival in patients with advanced HCC and inhibit migration of HCC cells [26, 27]. Hence, we examined the possible cooperative effects between sorafenib and MTBP overexpression on HCC migration. We chose 5 μM of sorafenib which had little effects on viability of both PLC/PRF/5 and Huh7 cells following 24 hours of treatment (Supplementary Figure 7A). Although sorafenib alone inhibited migration of PLC/PRF/5 and Huh7 cells, the inhibitory effects of sorafenib on the migration of MTBP-overexpressing cells were subtle without statistical significance (Supplementary Figure 7B). Thus, we could not detect obvious cooperative effects between sorafenib and MTBP overexpression in the experimental condition we used. These results further support our findings that sorafenib and MTBP, both commonly inhibit the Erk pathway but each has non-overlapping effects on cancer cell migration [7, 26].

## DISCUSSION

In this study, we have found that high MTBP expression in HCC cells and tissues are associated with increased cytoplasmic localization of p-Erk. This is caused by competitive binding of MTBP to IPO7, a p-Erk nuclear transporter. As a result, MTBP inhibits the p-Erk's ability to phosphorylate and activate Elk-1 transcription factor, leading to inhibition of HCC migration (Figure 7D). Activation of the MAPK/Erk signaling pathway is well characterized for regulation of HCC progression and metastasis via multiple molecular machineries [28–30]. Thus, our study has revealed a novel mechanism behind HCC migration and metastasis through the MTBP-IPO7-Erk1/2-Elk-1 axis and suggests that reduced MTBP could serve as a novel biomarker of HCC metastasis.

Due to lack of NLS in Erk1/2 and their molecular sizes (42 and 44 kDa), Erk1/2 was originally believed to be passively imported into the nucleus. Several specialized anchor/scaffold proteins that retain p-Erk in the cytoplasm have also been found [22]. Recent studies have identified IPO7 as a protein that actively binds to and imports p-Erk1/2 into the nucleus [24, 25]. Our results indicate that MTBP is a novel player that inhibits the p-Erk-IPO7 interaction as well as nuclear translocation of p-Erk. However, it is still unclear whether MTBP directly binds to IPO7 or if another protein mediates the MTBP-IPO7 interaction in cells.

Erk1/2 plays crucial roles in not only Elk-1 phosphorylation, but also mitochondrial biogenesis, Golgi fragmentation, and cytoskeletal reorganization [31]. Further studies are required to determine whether MTBP has any impact on these Erk1/2 functions. On the other hand, MTBP has also been reported to regulate other biological functions including replication origin firing [14], mitotic checkpoint [13], and c-Myc activity [10, 11]. It would be important to investigate potential involvement of Erk1/2 and IPO7 in these biological phenotypes regulated by MTBP and also determine whether or not MTBP regulates the nuclear translocation of some of the key factors involved in these cellular activities.

Elk-1 regulates migration of multiple cancer types through its downstream targets including *c-fos*, *EGR1*, and *PKCα* [32–34]. Specifically, PKCα is shown to be associated with HCC cell migration and invasion and is highly expressed in the poorly-differentiated human HCC cells [32, 35]. Also, both c-fos and EGR1 are implicated in promoting HCC migration and progression [36, 37]. Hence, it would be tempting to identify a novel and critical Elk-1 target that regulates HCC metastasis and to examine whether MTBP suppresses its expression.

Although MTBP has a NLS and is located to both the cytoplasm and the nucleus [7], the inhibitory effect of MTBP on p-Erk1/2 signaling is mainly caused by cytoplasmic MTBP, since a mutant MTBP^NLS^ still retains the abilities to interact with IPO7 and inhibit nuclear translocation of p-Erk1/2. This finding is in concordance with our previous report where cytoplasmic MTBP binds to ACTN4 and inhibits ACTN4-mediated migration of cancer cells including HCC [7, 9]. Thus, the cytoplasmic MTBP appears to suppress cancer cell migration via multiple mechanisms.

Our unbiased luciferase-based pathway analyses show that MTBP might regulate other signaling pathways or their downstream targets apart from the Erk1/2-Elk-1 signaling pathways. These include vitamin D receptor, MTF1, estrogen receptor, and HIF-1. Future studies are required to further validate these findings.

A compelling hypothesis is that MTBP may also regulate p-Erk nuclear import in multiple types of cancer cells. Since Erk1/2 activity is associated with disease progression and prognosis of HCC and other types of cancer [38–40], it would be vital to test this hypothesis. In conclusion, this study provides a new insight into HCC cell migration and metastasis through the regulation of the Erk1/2-Elk-1 pathway and helps identify a new therapeutic target for HCC metastasis.

## MATERIALS AND METHODS

### Cell lines

Huh7, PLC/PRF/5, and HepG2 cell lines were obtained from the KUMC liver center and maintained in Dulbecco's Modified Eagle's Medium (DMEM) with 10% fetal bovine serum (FBS) and 1% penicillin-streptomycin in a humidified incubator at 37^°^C with 5% CO_2_. All cell lines were authenticated in the University of Arizona Genetics Core facility. Cell were treated with EGF at 50 ng/ml for 15-30 min for examining RNA and protein expression, while 100 ng/ml of EGF was used for 12 hours of migration assays.

### Plasmids and shRNAs

Coding regions for *MTBP and ELK-1* were inserted into pCDH-CMV-MCS-EF1-Puro lentiviral vector (CD510B-1, SBI System Biosciences). FLAG-tagged MTBP^NLS^ and ΔC-MTBP were also inserted into the pCDH-CMV-MCS-EF1-Puro vector, while FLAG-tagged F4-MTBP was inserted into pEF/FRT/V5-D-TOPO vector (Invitrogen, Grand Island, NY, USA).

The following are shRNA-encoding lentiviral vectors (cat#, target sequence) purchased from sigma: human *Elk-1*-specific shRNA_1 (clone id-TRCNOT_042001_proof7450, CCGGCCCAAGAGTAACTCTCATTATCTCGAGATAATGAGAGTTACTCTTGGGTTTT), *Erk1* (MAPK3, clone id- TRCNOT_042001_proof6150, CCGGCCTGAATTGTATCATCAACATCTCGAGATGTTGATGATACAATTCAGGTTTT) and *Erk2* (MAPK1, clone id- TRCN0000010040, CCGGCAAAGTTCGAGTAGCTATCAACTCGAGTTGATAGCTACTCGAACTTTGTTTTT). Lentiviral vector encoding a non-target control (RHS4346) and a human *MTBP* shRNA (V2LHS_253815) were purchased from GE Dharmacon RNAi Technologies (Lafayette, CO, USA).

### Antibodies

For western blotting, the following antibodies were used: mouse monoclonal anti-MTBP (sc-137201, Santa Cruz), rabbit polyclonal anti-GAPDH (sc-27117, Santa Cruz), goat polyclonal anti-Lamin B (sc-6216, Santa Cruz), rabbit monoclonal anti-Elk-1 (# 9182, Cell Signaling), rabbit monoclonal anti-p-Elk-1 Ser383 (# 9181, Cell Signaling), rabbit monoclonal anti-Erk1/2 (# 4695, Cell Signaling), mouse monoclonal anti-p-Erk1/2 (# 9106, Cell Signaling), rabbit monoclonal anti-importin 7 (PAS5-25349, Thermo Scientific), mouse monoclonal anti-vinculin (10R-C105a, Fitzgerald, Acton, MA, USA). For co-immunoprecipitation studies; antibodies for mouse monoclonal anti-importin 7 (sc-271701, Santa Cruz), goat polyclonal anti-MTBP (sc-4717, Santa Cruz), rabbit monoclonal anti-p-Erk1/2 Thr202/Tyr204 (#4370, Cell Signaling), and mouse monoclonal anti-FLAG (M2, Sigma) were used. For immunofluorescence and IHC studies, antibodies for goat polyclonal anti-MTBP (N-13, sc-47174, Santa Cruz) and rabbit monoclonal anti-p-Erk1/2 Thr202/Tyr204 (#4370, Cell Signaling) were used.

### Immunofluorescence

Cells with or without treatment with EGF at 50 ng/ml for 30 min were plated onto poly-D-lysine/laminin-coated glass coverslips (BD Biosciences), fixed, and permeabilized with 4% formaldehyde in 100 mM PIPES (pH6.8), 10 mM EGTA, 1 mM MgCl_2_, and 0.2% Triton-X 100 for 15 min at room temperature. Following PBS washing, cells were blocked in 1% BSA in PBS plus 0.1% Tween 20 (PBS-T) for 30 min and further incubated with primary antibodies overnight at 4°C. After PBS-T washing, cells were incubated with the appropriate secondary antibodies. Samples were mounted in the ProLong Gold Antifade Reagent (Invitrogen), and results were analyzed with a Nikon epifluorescence microscope (Nikon).

### Signal pathway analysis

The Cignal Transduction 45-Pathway Reporter Array was purchased from SA Biosciences (Valencia, CA). Assays were performed according to the manufacturer's instructions. Luciferase activity was measured using The Dual-Glo® Luciferase Assay System (Promega, Madison, WI) with BioTek Synergy H4 multifunctional plate reader (BioTek).

### qRT-PCR

Total RNA from cell lines with or without treatment with EGF at 50 ng/ml for 15 min was isolated by using RNA Isolation Kit (Zymo research) and was reverse transcribed with Invitogen cDNA Synthesis Kit, starting with 1 μg total RNA from each sample, according to the manufacturer's instructions. For detecting mRNA expression of Elk-1 downstream target genes, assays for *EGR1* (Hs.PT.56a.40805543.g; IDT, Coralville, IA), *c-fos* (Hs.PT.56a.15540029; IDT), and *PKCα* (Hs.PT.56a.15189405; IDT), and *GAPDH* (Hs 9999905-m1, Life Technologies) were used and analyzed using Applied Biosystems ViiA7 (Life Technologies).

### Transwell migration assay

Migration assays were performed with 24-well transwell chambers (6.5mm diameter, 8 mm pore size, Corning). Cells (2×10^4^ for PLC/PRF/5, 1×10^4^ for Huh7, 1×10^4^ for HepG2) in 100 μl of 0.5% FBS-containing DMEM were seeded on the upper chamber, whereas in the lower chamber, 10% FBS-containing DMEM was added as a chemoattractant. EGF at 100 ng/ml was added into both the chambers. Cells were allowed for migrating through the membrane for 12 h. For the experiments with sorafenib (Santa Cruz; sc-220125), cells were pretreated with 5 μM of sorafenib for 12 hours, and sorafenib was continuously added into both the chambers during migration assays. The non-migrating cells were removed from the upper surface of the filters using cotton swabs and migrating cells to the lower surface were stained with Diff-Quik Stain Set (Dade Behring). Stained cells in the entire fields were counted under an inverted microscope.

### Co-immunoprecipitation

Cells were lysed with IP lysis buffer (Sigma) containing protease inhibitor cocktail (Roche Applied Science). Approximately 200 μg of whole cell lysates were incubated with protein-specific antibodies overnight at 4°C, followed by precipitation of the antibody-protein complex using protein A/G plus-agarose (Santa Cruz Biotechnology). 10% of the total amount of protein lysate (~20 μg) was used for input. In each experiment, an isotype negative control was used. After washing with IP lysis buffer, precipitates were analyzed by western blotting.

### Immunohistochemistry (IHC)

Liver cancer tissue array was purchased from US Biomax (LV1221) containing liver carcinoma and non-tumor liver tissues. Another 5 non-tumor liver tissues were obtained from the KUMC Liver Center to increase the number of controls. Tissue sections were deparaffinized in xylene and rehydrated through a series of graded alcohols. Slides were washed with PBS, and endogenous peroxidases were blocked with 1.5% hydrogen peroxide in PBS for 20 min at 25°C. After three 5 min washes with PBS, slides were incubated in blocking solution (PBS with 0.1% Triton X-100, 3% bovine serum albumin) with 5% normal donkey serum for 10 min at 25°C. Following antigen retrieval with sodium citrate buffer (10mM sodium citrate, pH 6.0) for 20 min, IHC was performed using the Vector R.T.U. Vectastain Kit (PK-7800). The sections were incubated with primary antibodies overnight at 4°C, washed with PBS, and were incubated with biotinylated secondary antibodies at room temperature for 30 min. Vector ImmPact DAB Peroxidase Substrate Kit (Vector Labs, Cat #SK-4105) was used for color development, followed by hematoxylin counterstaining. All stained sections were blindly evaluated by three independent investigators. Expression scores were determined based on intensity and extensity by assessing the whole tumor section. Each sample was scored on a scale of 0–3 for extensity with 0 corresponding to less than 25% of positive tumor cells; 1 for 26–50%; 2 for 51–75%; and 3 for 76–100% and the intensity of immunostaining was graded as 0 (negative staining), 1 (weakly positive staining), 2 (moderately positive staining), and 3 (strongly positive staining). The immunoreactive expression score of each section was calculated by the sum of these two parameters and presented as a score ranging between 0-6 as previously described [41]. Three independent investigators evaluated all stained sections. Final score was taken as an average from their individual score.

### Statistical analysis

Experiments were performed independently at least three times with values expressed as mean ± standard deviation (S.D.). For IHC analyses, a Kruskal-Wallis test was used to compare MTBP levels across non-tumor liver, primary HCC and metastatic HCC tissues. Pairwise comparisons were conducted with 1-tailed Mann-Whitney tests, using a nominal significance level of 0.05 and considering p-values <0.017 as significant for a Bonferroni correction. Association between p-Erk localization (C>N vs C≤N) and MTBP expression score (<3 vs ≥3) was investigated with a Fisher's exact test in a bivariate analysis. To control the potentially confounding effect of tumor location, an exact logistic regression of p-Erk localization was used; the dependent variable of the model was p-Erk localization, while the independent variables were MTBP categorization and tumor location. All other experimental results were analyzed using Student's *t-*test. In these analyses, a p-value of 0.05 or lower was considered to be statistically significant. Statistical analyses were performed with Graph Pad Prism software (San Diego, CA) and Stata (StataCorp LP, College Station, TX).

## SUPPLEMENTARY MATERIALS FIGURES AND TABLES



## Abbreviations

MDM2: mouse double minute 2 homolog
MTBP: MDM2 Binding Protein
ACTN4: α-actinin 4
Erk: extracellular signal-regulated kinase
MAP: mitogen-activated protein kinase
RT-PCR: reverse transcriptase-polymerase chain reaction
PBS: phosphate buffered saline
RIPA: radio immunoprecipitation assay
DMSO: dimethyl sulfoxide
*EGR-1*: early growth response protein 1
PKCα: protein kinase C alpha
