# Supplementary material for: MTBP inhibits the Erk1/2-Elk-1 signaling in hepatocellular carcinoma
Source: Oncotarget. 2018 Apr 20;9(30):21429–43. doi: 10.18632/oncotarget.25117 (PMC5940416; doi:10.18632/oncotarget.25117)
Supplement: Supplementary file 1 [file oncotarget-09-21429-s001.pdf]

MTBP inhibits the Erk1/2-Elk-1 signaling in hepatocellular carcinoma

SUPPLEMENTARY MATERIALS

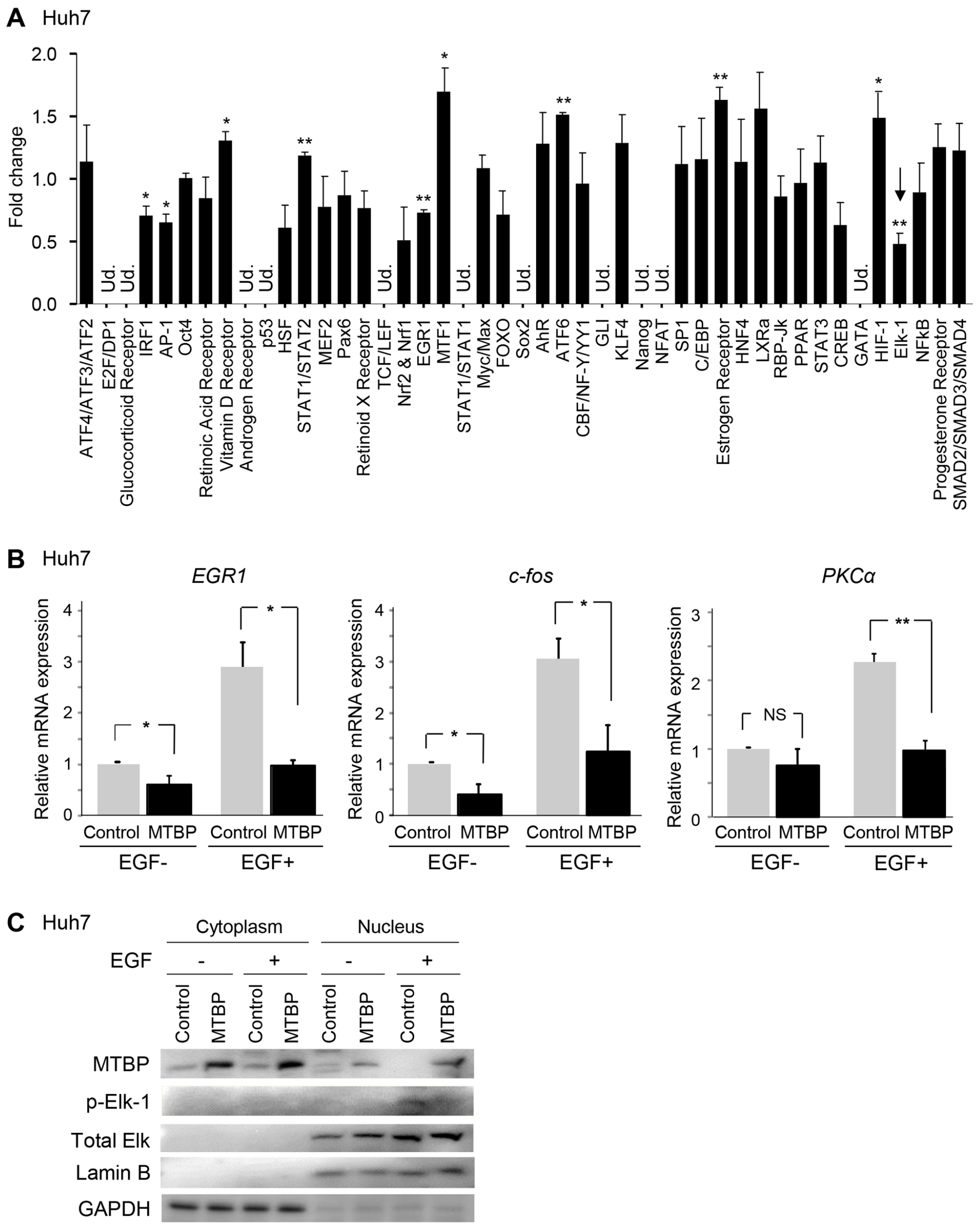

Supplementary Figure 1: (A) Luciferase-based signal array analysis using Huh7 cells infected with lentiviral vectors encoding empty (control) or MTBP cDNA. Results showing fold change of the luciferase activity altered by MTBP overexpression, compared to the control lentiviral vector (set as 1) of 45 genes related different signaling pathways. An arrow indicates that Elk-1 activity was significantly suppressed by MTBP overexpression. Ud., undetectable. Error bars: mean ± S.D. (n=3 independent experiments). Student's *t* test: *, $P < 0.05$ and **, $P < 0.01$. Other pathways had no statistical significance. **(B)** Results of qRT-PCR for *EGR1*, *c-fos*, and *PKCα* using Huh7 cells infected with lentiviral vectors encoding empty (control, grey) or MTBP cDNA (black), with (+) or without (-) 50 ng/ml of EGF stimulation for 15 min. Data are normalized by values of *GAPDH* mRNA. Error bars: means ± S.D. from three independent experiments. Student's *t* test: *, $P < 0.05$ and **, $P < 0.01$. NS, not significant. **(C)** Western blotting for MTBP, p-Elk-1 at serine 383, total Elk-1, Lamin B, and GAPDH using cytoplasmic and nuclear protein extracts from Huh7 cells treated with solvent (-) or 50 ng/ml of EGF (+) for 30 min.

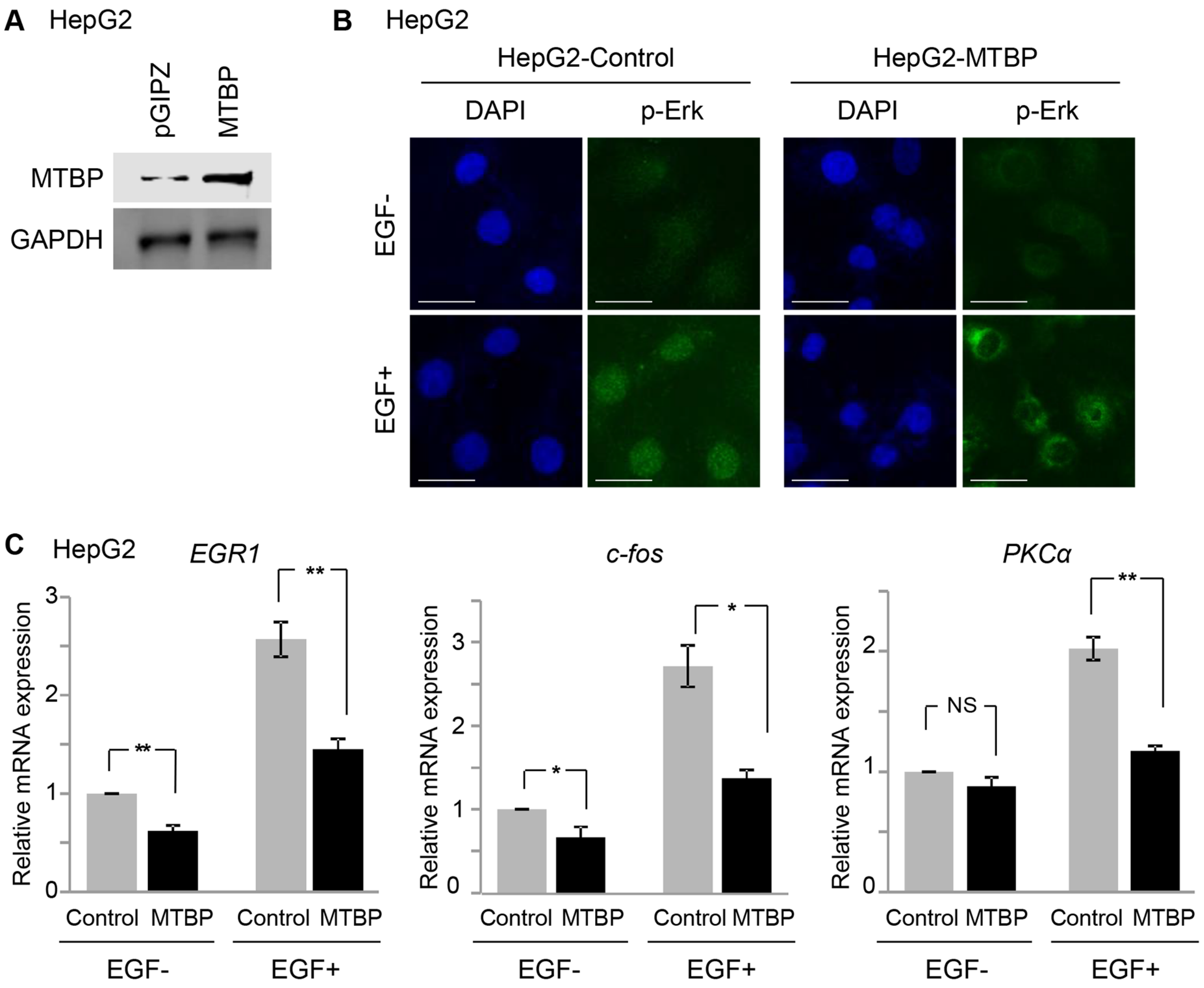

Supplementary Figure 2: **(A)** Western blotting for MTBP and GAPDH, showing overexpression of MTBP in HepG2 (wild-type p53) cells infected with lentiviral vectors encoding non-target (control) or MTBP cDNA (MTBP). **(B)** Immunofluorescence studies for DAPI and p-Erk, following treatment of control or MTBP-overexpressing HepG2 cells with (+) or without (-) 50 ng/ml of EGF treatment for 30 min. Scale bar, 50 μm. **(C)** Results of qRT-PCR for *EGR1*, *c-fos*, and *PKCα* using HepG2 cells infected with lentiviral vectors encoding empty (control, grey) or MTBP cDNA (black), with (+) or without (-) 50 ng/ml of EGF stimulation for 15 min. Data are normalized by values of *GAPDH* mRNA. Error bars: means ± S.D. from three independent experiments. Student's *t* test: *, $P < 0.05$ and **, $P < 0.01$. NS, not significant.

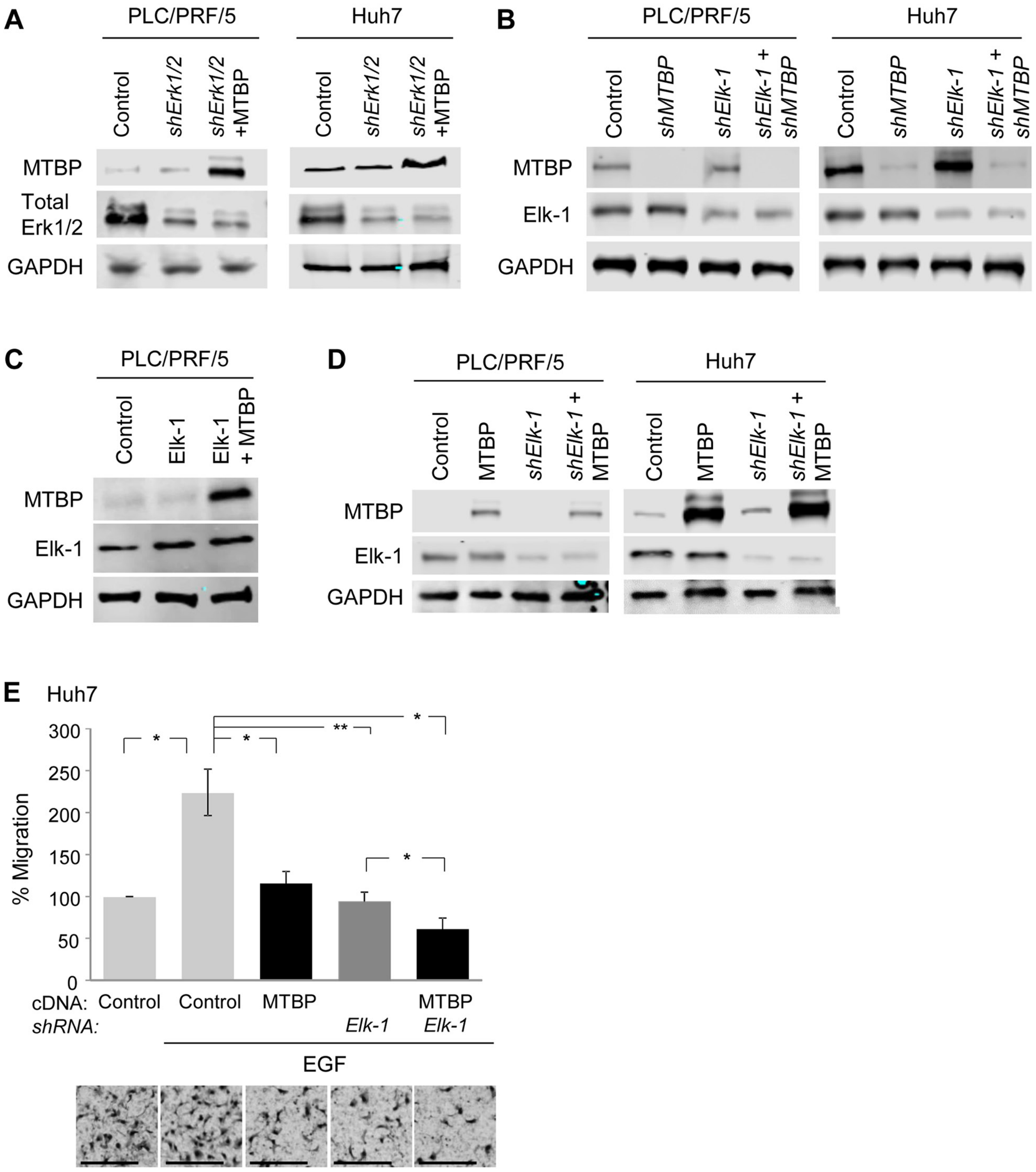

Supplementary Figure 3: **(A)** Western blotting showing knockdown of Erk1/2 and overexpression of MTBP in both PLC/PRF/5 (left) and Huh7 (right) cells infected with non-target shRNA (control), shRNA for Erk1/2 (*shErk1/2*) and/or MTBP cDNA (MTBP). **(B)** Western blotting for MTBP, Elk-1 and GAPDH in PLC/PRF/5 (left) and Huh7 (right) cells following knockdown of MTBP and/or Elk-1. **(C)** Western blotting for MTBP, Elk-1, and GAPDH in PLC/PRF/5 cells with overexpression of MTBP and/or Elk-1. **(D)** Western blotting showing knockdown of Elk-1 (*shElk-1*) and/or overexpression of MTBP (MTBP) in PLC/PRF/5 (left) and Huh7 (right) cells. **(E)** Transwell migration assays using Huh7 cells infected with non-target (control) or *Elk-1* (*Elk-1*) shRNAs with or without overexpression of MTBP, in the presence of 100 ng/ml of EGF. Graphs showing relative cell migration (%) compared to the number of migrating cells in control (left). Representative images below the graphs. Error bars: means ± S.D. from three independent experiments. Student's *t* test: *, $P < 0.05$ and **, $P < 0.01$. Scale bar, 25 μm.

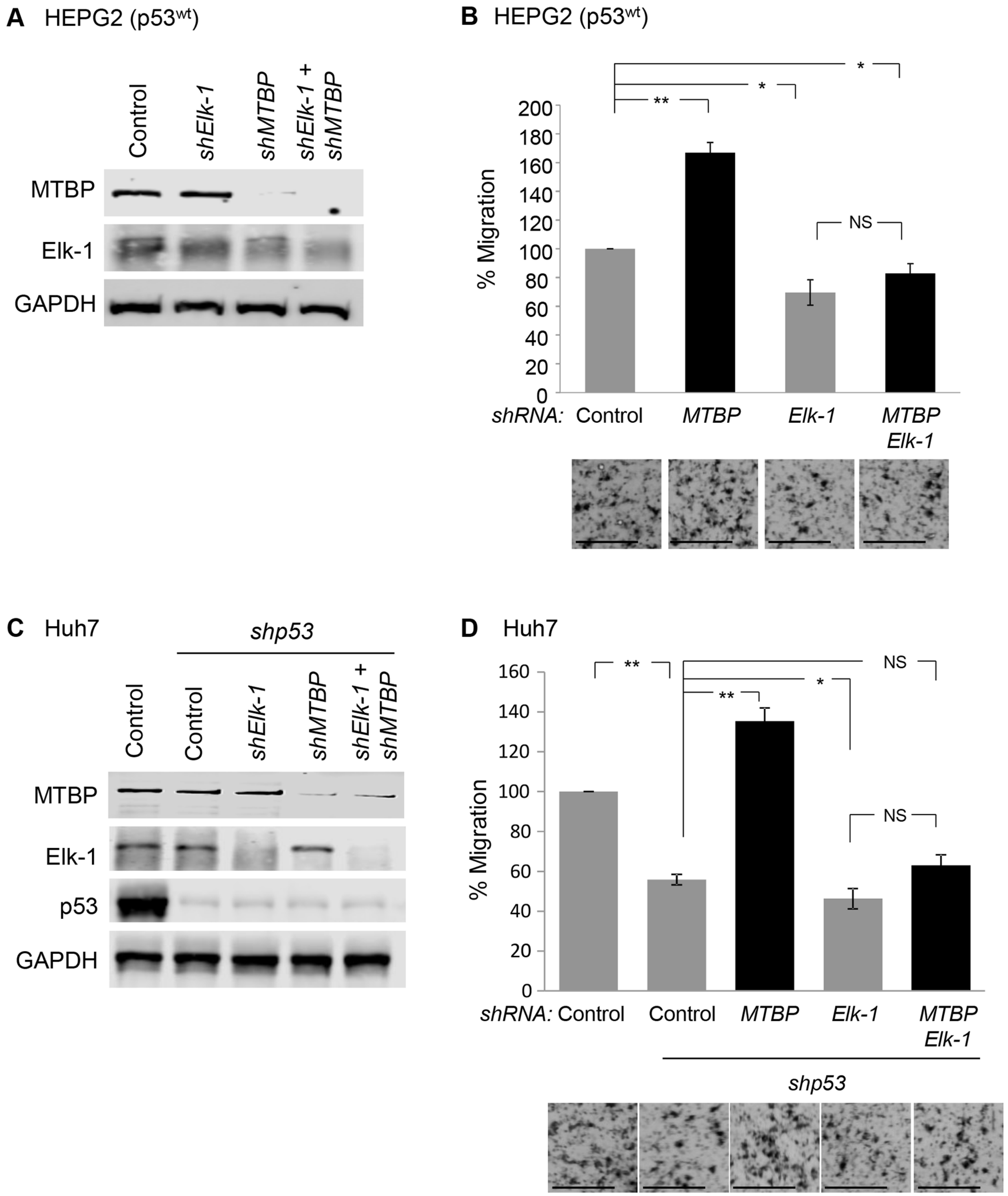

Supplementary Figure 4: (A) Western blotting for, MTBP, Elk-1, and GAPDH in HepG2 cells following knockdown of MTBP (*shMTBP*) and/or Elk-1 (*shElk-1*). **(B)** Transwell migration assays for 12 hours using HepG2 cells with or without knockdown of MTBP (*MTBP*) and/or Elk-1 (*Elk-1*). Representative images below the graphs. **(C)** Western blotting for MTBP, Elk-1, p53, and GAPDH in Huh7 cells with or without p53 knockdown (*shp53*), together with knockdown of MTBP (*shMTBP*) and/or Elk-1 (*shElk-1*). **(D)** Transwell migration assays for 12 hours using Huh7 and p53-knockdown (*shp53*) Huh7 cells with or without MTBP (*MTBP*) and/or Elk-1 (*Elk-1*) knockdown. Representative images below the graphs. Error bars: means ± S.D. from three independent experiments. Student's *t* test: *, $P < 0.05$ and **, $P < 0.01$. NS, not significant. Scale bar, 25 μm.

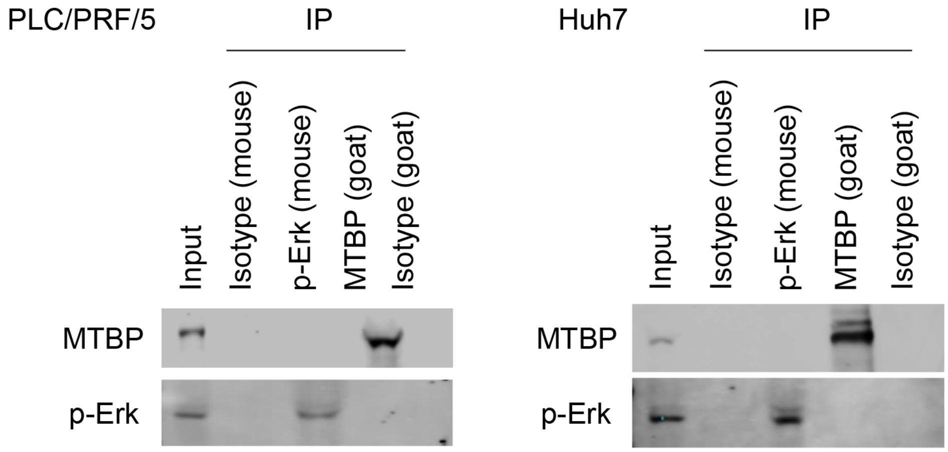

Supplementary Figure 5: Co-immunoprecipitation (IP) studies for endogenous p-Erk and MTBP using protein extracts (~200 µg) from PLC/PRF/5 (left) and Huh7 (right) cells. Isotypes were used as negative controls. 10% of the total amount of protein lysate (~20 µg) was used for input.

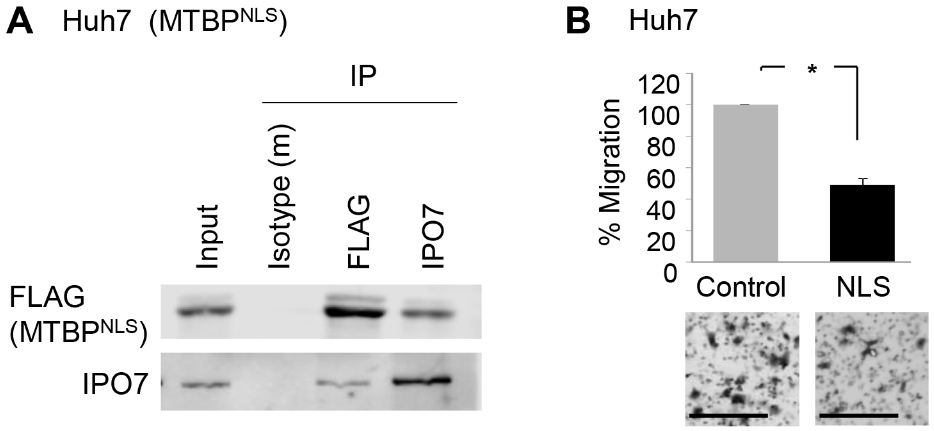

Supplementary Figure 6: (A) Co-immunoprecipitation studies between a mutant MTBP with disrupted NLS ($MTBP^{NLS}$) and IPO7 using protein lysates (~200 µg) from Huh7 cells expressing FLAG-tagged $MTBP^{NLS}$. $MTBP^{NLS}$ was precipitated using anti-FLAG M2 affinity gel. Isotypes were used as negative controls. 10% of the total amount of protein lysate (~20 µg) was used for input. **(B)** Transwell migration assays using Huh7 cells infected with lentiviral vectors encoding empty (control) or FLAG-tagged $MTBP^{NLS}$ (NLS). Graphs showing relative cell migration (%) compared to the number of migrating cells in the control. Error bars: means ± S.D. from three independent experiments. *, Student's *t* test: *, $P < 0.05$.

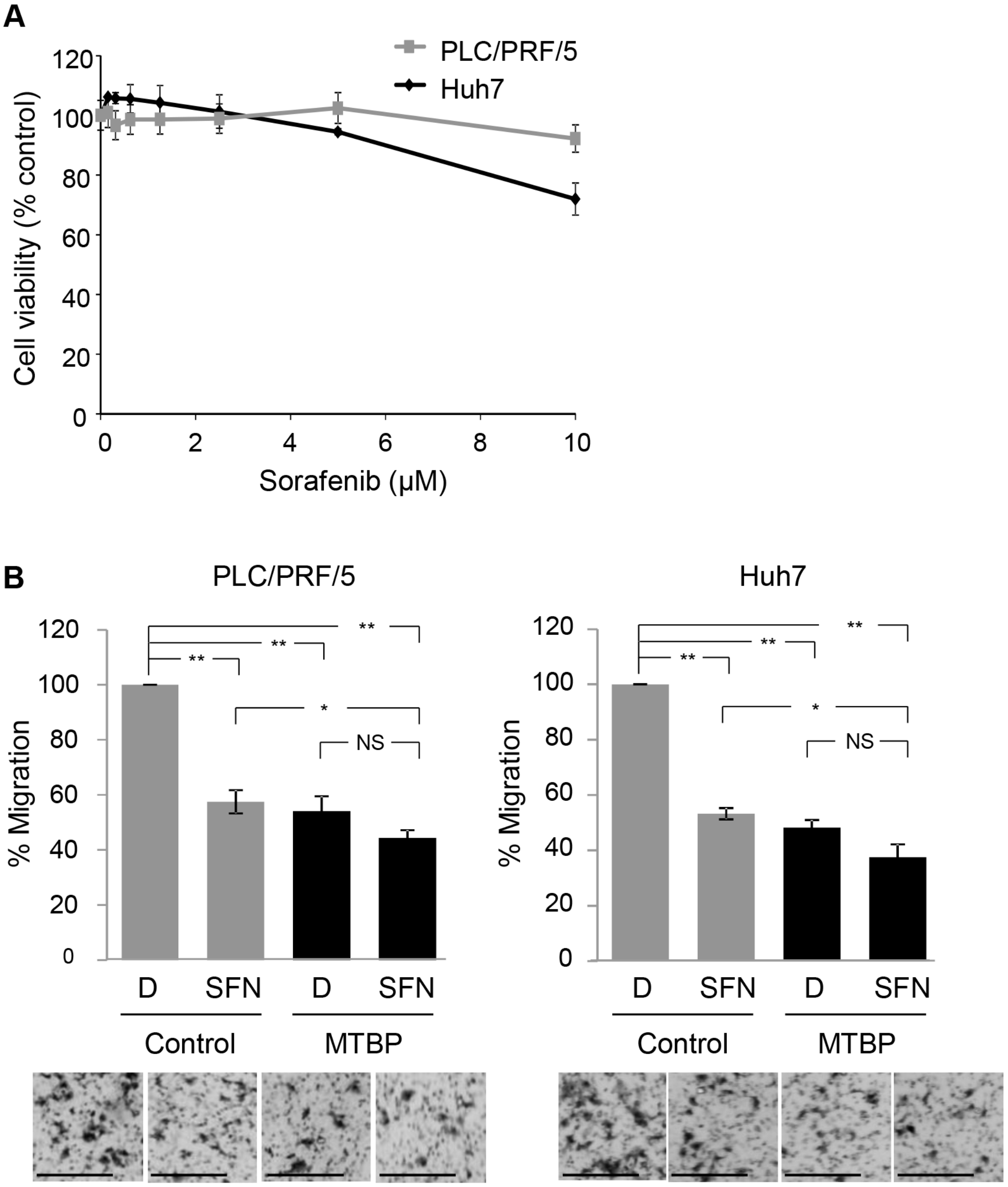

Supplementary Figure 7: (A) Examination for the effects of sorafenib on viability of PLC/PRF/5 (grey) and Huh7 (black) cells using MTT assays. Cells (5,000) were seeded onto a 96-well plate. Twenty four (24) hours later, cells were treated with varying concentrations of sorafenib (0-10 μM) for 24 hours, followed by MTT assays according to the manufacture's instruction (Fisher Scientific, M6494). Error bars: means ± S. D. from three independent experiments. **(B)** PLC/PRF/5 (left) and Huh7 (right) cells with or without MTBP overexpression were pre-treated with DMSO (D) or 5 μM of sorafenib (SFN) for 12 hours before the transwell migration assays. Following trypan-blue staining, viable cells were subjected to the migration assay for 12 hours. Both upper and lower chambers were supplemented with DMSO (D) or sorafenib (SFN, 5 μM) during the assay. Graphs showing relative cell migration (%) compared to the number of migrating cells in control. Error bars: means ± S.D. from three independent experiments. Student's *t* test: *, $P < 0.05$, **, $P < 0.01$; NS, not significant.

Supplementary Table 1: Summary of immunohistochemistry (IHC) for MTBP and p-Erk

HCC location	Cases	MTBP score		p-Erk score		p-Erk localization		p-Erk localization Both primary and Metastasis (p=0.034)[a]	
Primary	57	41	16	30	27	N≥C 17<br>C>N 6<br>UD 18	N≥C 6<br>C>N 8<br>UD 2	N≥C 28<br>C>N 18<br>UD 28	N≥C 6<br>C>N 11<br>UD 2
Metastasis	36	33	3	24	12	N≥C 11<br>C>N 12<br>UD 10	N≥C 0<br>C>N 3<br>UD 0		

N: nuclear staining. C: cytoplasmic staining. UD: undetermined.

[a]p-value testing the association between p-Erk localization (C>N vs N≥C) and MTBP score (<3 vs ≥3), adjusting for tumor location, according to an exact logistic regression. Before adjusting for tumor location, the association was not significant (p=0.13).

Supplementary Table 2: Correlation of MTBP or p-Erk with size and pathological grade of primary tumors

Size (cm)	Cases	MTBP score		p-Erk score	
		**<3**	**≥3**	**<3**	**≥3**
<5	18	14	4	10	8
≥5	39	27	12	20	19
		N.S.		N.S.	
**Pathological grade**	**Cases**	**MTBP score**		**p-Erk score**	
		**<3**	**≥3**	**<3**	**≥3**
1	4	3	1	2	2
2	37	26	11	16	21
3	8	4	4	5	3
NA	8	8	0	7	1
		N.S.		N.S.	

NA: not applicable. N.S.: not significant. Fisher's exact test.
